# Two-Dimensional Shear Wave Elastography of Normal Soft Tissue Organs in Adult Beagle Dogs; Interobserver Agreement and Sources of Variability

**DOI:** 10.3389/fbioe.2020.00979

**Published:** 2020-08-19

**Authors:** Jin-Woo Jung, Hyejin Je, Sang-Kwon Lee, Youjung Jang, Jihye Choi

**Affiliations:** College of Veterinary Medicine and BK21 Plus Project Team, Chonnam National University, Gwangju, South Korea

**Keywords:** dog, elastography, reproducibility, two dimensional, shear wave

## Abstract

Shear wave elastography (SWE) induces lateral shear wave through acoustic pulses of the transducer and evaluates tissue stiffness quantitatively. This study was performed to evaluate feasibility and reproducibility of two-dimensional shear wave elastography (2D SWE) for evaluation of tissue stiffness and to examine technical factors that affect shear wave speed (SWS) measurements in adult dogs. Nine healthy, 2 year-old, adult beagles with the median weight of 9.8 kg were included. In this prospective, experimental, exploratory study, 2D SWE (Aplio 600) from the liver, spleen, kidneys, pancreas, prostate, lymph nodes (submandibular, retropharyngeal, axillary, medial iliac, and inguinal), submandibular salivary gland, and thyroid was performed in anesthetized beagles. Color map was drawn and SWS of each SWE were measured as Young’s modulus (kPa) and shear wave velocity (m/s). The effect of measuring site, scan approach, depth, and anesthesia on SWE was assessed in abdominal organs by two observers independently. A total of 27 SWE examinations were performed in 12 organs by each observer. All SWS measurements were preformed successfully; however, SWE in the renal medulla could not be successfully conducted, and it was excluded from further analysis. Interobserver agreement of SWE was moderate to excellent in all organs, except for the left liver lobe at 10–15 mm depth with the intercostal scan. In the liver, there was no significant effect of the measuring site and scan approach on SWE. SWS of the liver and spleen tended to be higher with increasing the depth, but no significant difference. However, anesthesia significantly increased tissue stiffness in the spleen compared to awake dog regardless of the depth (*P* < 0.05). There was a significant difference in SWS according to the measuring site in the kidneys and pancreas (*P* < 0.001). 2D SWE was feasible and highly reproducible for the estimation of tissue stiffness in dogs. Measuring site and anesthesia are sources of variability affecting SWE in abdominal organs. Therefore, these factors should be considered during SWS measurement in 2D SWE. This study provides basic data for further studies on 2D SWE on pathological conditions that may increase tissue stiffness in dogs.

## Introduction

Stiffness indicates tissue resistance to deformation and can be expressed as deformability, which is the degree of deformation when a force is applied to the tissue (Shiina et al., 2015). Shear wave elastography (SWE) induces lateral shear wave through acoustic pulses of the transducer and evaluates tissue stiffness quantitatively with shear wave speed (SWS; Shiina et al., 2015; Sigrist et al., 2017). Two SWE methods are used; point shear wave elastography (pSWE); and two-dimensional elastography (2D SWE; Shiina et al., 2015; Dietrich et al., 2017; Sigrist et al., 2017; Zelesco et al., 2018).

In pSWE, the transducer generates a focal single acoustic pulse to induce displacement of tissue within a fixed region of interest (ROI; Cosgrove et al., 2013; Shiina et al., 2015; Sigrist et al., 2017). The generated shear wave is detected by multiple tracking beams positioned laterally and the average time to peak of the shear wave within ROI is calculated (m/s). The operator chooses the location of ROI for pSWE as the uniform area of the parenchyma, however, the generated shear wave is not visualized itself and ROI size is usually small and fixed (Cosgrove et al., 2013; Sigrist et al., 2017).

Two-dimensional shear wave elastography induces shear wave in multifocal zones and measures the SWS that propagates at several locations around the acoustic pulse through a detection pulse. Subsequently, SWS inside the field of view (FOV) is integrated and the distribution of SWS is displayed through a color map showing areas of high stiffness as red and low stiffness as blue (Cosgrove et al., 2013; Shiina et al., 2015; Barr et al., 2015a; Sigrist et al., 2017). The map can determine whether shear wave propagation within FOV is reliable. Proper induction of shear wave in normal organ is represented by a uniform blue color in FOV (Zelesco et al., 2018). The average SWS is also quantified as Young’s modulus in kilopascals (kPa) and shear wave velocity (SWV) measured in m/s by placing the ROI within FOV (Cosgrove et al., 2013; Shiina et al., 2015; Sigrist et al., 2017).

Two-dimensional shear wave elastography has been used mainly in diffuse liver disease for staging liver fibrosis in human medicine. Moreover, there are many studies about the 2D SWE application for differentiating between benign and malignant breast masses, differentiate between benign and malignant thyroid nodules, identification of the parathyroid adenoma and hyperplasia, and estimation of the kidney stiffness in chronic renal diseases (Barr et al., 2015b; Cosgrove et al., 2017; Golu et al., 2017; Lee et al., 2017; Bob et al., 2018; Kyriakidou et al., 2018; Liu et al., 2018; Cotoi et al., 2019; Grosu et al., 2019; Sãftoiu et al., 2019; Cotoi et al., 2020). In veterinary literature, 2D SWE was applied to distinguish malignant from benign conditions of mammary tumors and lymph nodes and to evaluate the margination and acute changes in stiffness of the ablated liver tissue after radiofrequency ablation in dogs (Glińska-Suchocka et al., 2013; Feliciano et al., 2017; Lee et al., 2018; Silva et al., 2018). Recently, 2D SWE confirmed that the SWS in dogs with clinically relevant hepatic fibrosis was significantly higher compared to those in healthy dogs and dogs without clinically relevant hepatic fibrosis (Tamura et al., 2019b). In addition, 2D SWE revealed the different SWS in dogs with suspected pancreatic diseases and clinically healthy dogs (Avante et al., 2020).

Shear wave elastography can be affected by various factors. Their effect on reliability and reproducibility of pSWE and 2D SWE according to the organs applied has been investigated in humans and guidelines for liver, breast, thyroid, and prostate elastography with 2D SWE have been established for proper shear wave induction (Cosgrove et al., 2013; Ferraioli et al., 2015; Barr et al., 2015a, b; Cosgrove et al., 2017; Dietrich et al., 2017; Sigrist et al., 2017; Zelesco et al., 2018). The guidelines suggest specific recommendations about the patient’s factors such as fasting, position, access, and breathing and also the technical factors such as depth, location, and size of ROI and the number of measurements for estimating SWS to perform the SWE for liver accurately (Ling et al., 2013; Ferraioli et al., 2015; Dietrich et al., 2017; Lee et al., 2017; Nadebaum et al., 2018; Zelesco et al., 2018; Gress et al., 2019). For the prostate elastography, minimal preload, the location of focus zone, FOV size, lesion location within the FOV box, and ROI range for encompassing the lesion, are also recommended (Barr et al., 2017; Sang et al., 2017). Besides, pressure and angle of the probe on the surface can induce false hardness as indicated by the red color on the color map, leading to false tumor diagnosis (Shiina et al., 2015; Barr et al., 2015a; Sigrist et al., 2017; Zelesco et al., 2018). Image quality of B-mode ultrasound, the measuring site, scan approach, and depth of the ROIs influence SWE in human studies (Ling et al., 2013; Dietrich et al., 2017; Nadebaum et al., 2018; Zelesco et al., 2018). However, in veterinary medicine, differences in SWS according to ROI depth and measuring site were investigated only for pSWE (Holdsworth et al., 2014). A recent study (Tamura et al., 2019a) assessed repeatability and reproducibility of 2D SWE in the liver and spleen and provided the SWS of the liver and spleen in healthy dogs. Meanwhile, the effect of the technical factors on measuring SWS such as ROI depth, measuring sites and approach, anesthesia has not been evaluated intensively and fundamental data of soft tissue organs including lymph nodes are not intensively investigated using 2D SWE in dogs.

The objectives of this study were to assess the feasibility of 2D SWE in soft tissue organs and to evaluate the effect of the technical factors on the SWS in adult dogs. We hypothesized that stiffness of canine organs can be determined using 2D SWE with reliable reproducibility and the SWS can be affected by the measuring sites, scan approach and the depth of ROIs.

## Materials and Methods

### Animals

In this prospective, experimental, exploratory study, nine healthy, and intact purpose-bred beagles (seven males, two females) were used. The median age of beagles was 2 years (1–5 years) and the median weight was 9.8 kg (8.5–12.5 kg). All dogs were clinically healthy based on physical examination, blood pressure, complete blood count, serum biochemistry, electrolyte concentration, urinalysis, abdominal radiography, ultrasonography, and echocardiography. Decisions for inclusion or exclusion of dogs were made by two veterinarians (J.W.J., H.J.J.) with 2 years of radiology experience. Dogs were housed individually and fed a commercial dry food and water *ad libitum.* This study protocol was approved by the Institutional Animal Care and Use Committee of Chonnam National University (CNU IACUC-YB-2018-90).

### SWE Examination of Each Organ

After fasting for at least 12 h, conventional ultrasonography and SWE were conducted using ultrasound scanner (Aplio I 600, Canon medical system, Tustin, United States) with a 5.0–14.0 MHz linear-array transducer (PLT-1005BT, Canon medical system). SWE was performed by two veterinarians (J.W.J., H.J.J.) with 7 years and 2 years of radiology experience, respectively, under the supervision of a radiologist (J.H.C). They performed SWE individually using the same ultrasound machine with an identical protocol in the same dog at a different day with at least a 3-day interval in random order. Both ultrasonography examinations were performed after clipping of the hair over the abdomen and cervix and applying ultrasonic gel to the skin. SWE performed according to the method used in previous studies and the recommended guidelines for the clinical use of elastography in humans (Cosgrove et al., 2013; Ferraioli et al., 2015; Shiina et al., 2015; Barr et al., 2015b, 2017; Cosgrove et al., 2017; Tamura et al., 2019a).

The examined areas included abdominal organs (liver, spleen, kidney, pancreas, and prostate), lymph nodes (medial iliac, inguinal, axillary, submandibular, and retropharyngeal lymph nodes), salivary gland, and thyroid. The effects of the technical factors on SWS were assessed about the measuring site, approach, and ROI depth in the liver, ROI depth and anesthesia in the spleen, and measuring site in the renal cortex, pancreas and prostate (Table 1).

**TABLE 1:** Technical factors in the abdominal organs in two-dimensional shear wave elastography.

	Technical factors				
	
					Number of SWE
Organ	Measuring site	Approach	Depth	Anesthesia	measurements
Liver	Left vs. right lobe	Intercostal vs. subcostal	5–10 mm vs. 10–15 mm	–	8
Spleen	–	–	0–5 mm vs. 5–10 m	Before vs. immediately after anesthesia vs. during recovery	6
Kidney	Left vs. right	–	–	–	2
Pancreas	Pancreatic body vs. right lobe	–	–	–	2
Prostate	Left vs. right lobe	–	–	–	2

**FIGURE 1:**
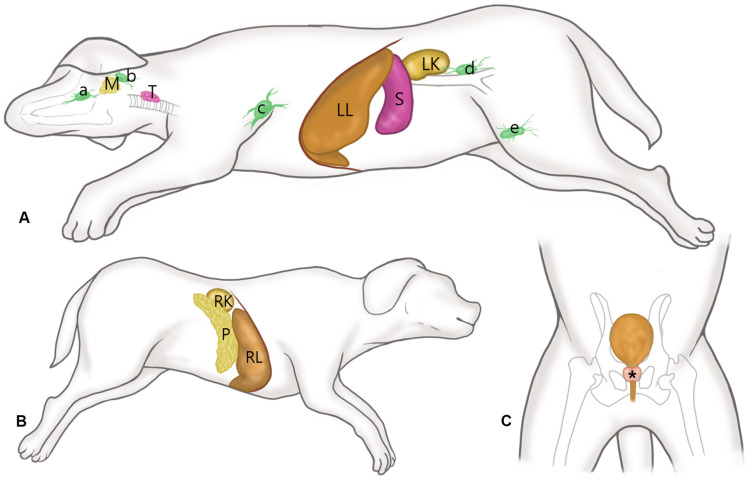
Schematic representations of the canine anatomy with indication of the examined soft tissue organs. **(A)** After placing the dogs in right lateral recumbency, shear wave elastography (SWE) of the submandibular (a), retropharyngeal (b), axillary (c), inguinal (d) and medial iliac (e) lymph nodes, submandibular salivary gland (M), thyroid (T), spleen (S), left liver lobe (LL), and left kidney (LK) was performed in the left side for each dog, while maintaining the vertical angle of the probe to the organ. **(B)** With the dog positioned in left lateral recumbency, SWE of the pancreas (P), right liver lobe (RL), and right kidney (RK) were performed. **(C)** SWE in the prostate (*) was conducted in the right and left lobes in the transverse images with the dog in dorsal recumbency.

The dog’s position for SWE is presented in Figure 1. After placing the dogs in right lateral recumbency, SWE of the submandibular, retropharyngeal, axillary, inguinal and medial iliac lymph nodes, submandibular salivary gland, thyroid, spleen, left liver lobe, and left kidney was performed in the left side for each dog. For imaging the axillary lymph node, the left forelimb was abducted and the lymph node was scanned immediately posterior to the division of brachial and subscapular vessels. SWE of the inguinal lymph node was performed with the left hindlimb pulled caudally. SWE of the right liver lobe, right kidney, and pancreas were performed with placing the dog in left lateral recumbency. SWE in the prostate was conducted in the right and left lobes in the transverse images with the dog in dorsal recumbency.

Shear wave elastography of the spleen was performed via a subcostal approach with placing the ultrasound probe under the ribs. SWS measurements were acquired at two separate, predefined depths (0–5 mm vs. 5–10 m) from the splenic body. SWE examinations of the spleen were performed three times in each dog; before anesthesia, immediately after anesthesia and during recovery (60 min after anesthesia induction) in each dog. Anesthesia was performed using intramuscular injection with a combination of 0.75 mg/kg zolazepam hydrochloride-tiletamine hydrochloride (Zoletil^®^, Virbac, Carros, France) and 0.03 mg/kg medetomidine hydrochloride (Domitor^®^, Orion Corporation, Espoo, Finland).

**FIGURE 2:**
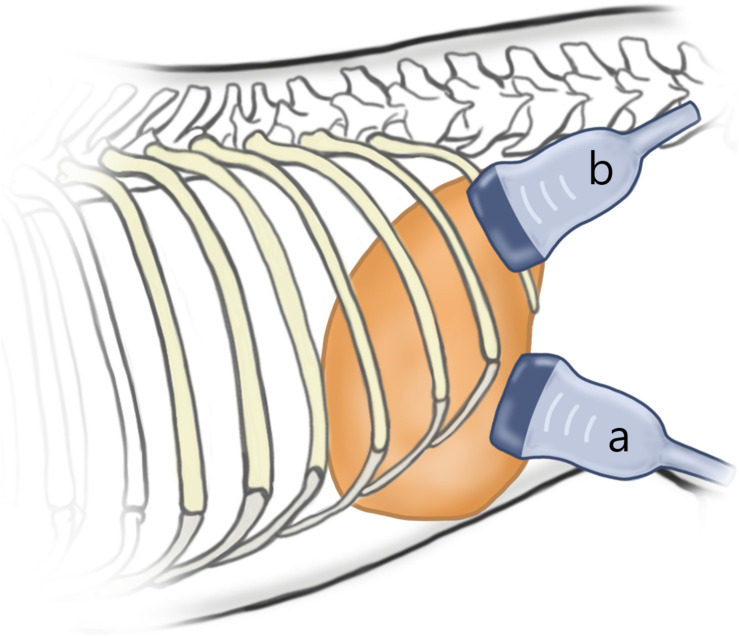
Schematic representation of the probe position according to the approach. For subcostal approach **(a)**, the ultrasound probe is placed under the ribs. For intercostal approach **(b)**, the probe was placed parallel to and within the intercostal space, and sufficient gel was applied to minimize rib shadowing.

Then, SWE in all other organs was performed under anesthesia. In the liver, SWE was performed totally eight times in each dog according to the two different measuring sites (left vs. right lobe), two different approaches (intercostal vs. subcostal), and two different depths from the liver capsule (5–10 mm vs. 10–15 mm). For intercostal approach, the probe was placed parallel to and within the intercostal space, and sufficient gel was applied to minimize rib shadowing (Figure 2). For a subcostal approach, the ultrasound probe was placed under the ribs. If gas in the duodenum, colon, or lung hindered imaging of right liver lobe, the probe was positioned more dorsally.

Shear wave elastography of the left and right kidney was performed from the renal cortex and medulla through the subcostal approach. If the sagittal image of each kidney could not be obtained without probe compression, the intercostal approach with the same method for the liver. SWE in the pancreas was performed from the pancreatic body and right lobe via subcostal approach.

### SWE Procedure

In each organ, the B-mode image was obtained from a region without large vascular and ductal structures, while maintaining the probe oriented perpendicular to the organ without compression. Intrusion depth was set at 4 cm in the abdomen and 2.5 cm in the lymph nodes, submandibular salivary gland, and thyroid. After scanning the target organ, a rectangular FOV was created over the B-mode image by using the installed 2D SWE software (Shear Wave Elastography version 6.0, Canon medical system). When the size of the target organ exceeded 2 cm (i.e., liver and spleen), a 3 × 3 cm FOV size was used. If the target organ was less than 2 cm, FOV size was adjusted to fill the target organs at least 80% in the FOV.

**FIGURE 3:**
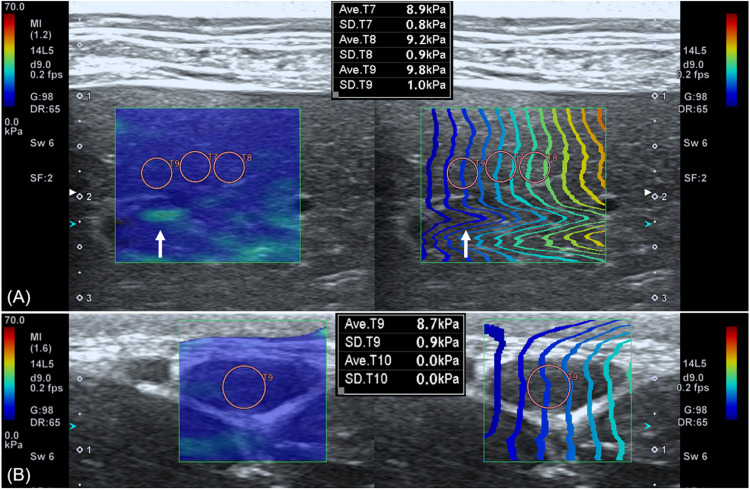
Shear wave elastography in the liver **(A)** and submandibular lymph node **(B)**. A dual-screen mode shows a color map on the left side and propagation map on the right of the view (rectangular boxes). On color maps, red color represents high shear wave velocity and blue low velocity. Propagation maps show multiple contour line depicting shear wave arrival time at different location. **(A)** In the liver, circular regions of interest (ROIs) with 3 mm diameter are placed over the region where uniform blue color on color map and parallel contour lines with constant interval on propagation map are observed. Care was taken not to place ROI over the region (arrow) displayed as green color in the color map and distorted contour line in the propagation map. **(B)** In the submandibular lymph node, a 2 mm diameter ROI not to exceed the target organ is used.

Push pulse was emitted at the end-expiration to reduce the respiratory motion artifact. After image acquisition, the color and propagation maps were displayed on dual-screen mode, side by side. In the color map, the relative stiffness, high, intermediate, or low stiffness, was color-coded in red, green, or blue, respectively. The appearance of a block spot inside the color map indicated an area where SWS cannot be measured, because tissue was not vibrating enough or the amplitude of shear wave was too low (Cosgrove et al., 2013). Propagation map displayed contour lines to depict shear wave arrival times at different locations, illustrating shear wave properties to be generated within FOV. An area with a constant interval between contour lines and without distortion suggests that SWS is constant without artifacts (Zelesco et al., 2018). ROI placement was optimized inside the FOV based on the color and propagation maps (Nadebaum et al., 2018). The SWS was measured by placing the circular ROIs over parenchyma (bulk of functional substance in an animal organ) in FOV, where both uniform blue color without black spots on color map and parallel contour lines with constant intervals on the propagation map had appeared (Figure 3). In general, ROI with 3 mm diameter was used and if the size of ROI exceeded the target organ, ROI was adjusted to 2 mm. Less than 3 ROIs were placed in each FOV without overlapping to each other. In each ROI, the SWS were recorded in Young’s modulus (kPa) and SWV (m/s). Young’s modulus (kPa) was converted from the SWV (m/s) using the relationship E = 3ρc_s_^2^ in which ρ represents tissue density, and c_s_ represents SWV by the installed software in the ultrasound machine automatically and tissue density was assumed to be 1 g/cm^3^.

Shear wave elastography was performed by repeating the placement of FOV and ROIs at different locations. Data were measured in mean and standard deviation (SD) by two veterinarians (J.W.J., H.J.J.) independently blinded to each other’s measurements. From each study, measurements were repeated until five data were selected for further analysis (Zelesco et al., 2018). Data was selected when the SD was less than 15% of the mean value by following the more strictly modified guideline for human elastography (Barr et al., 2015a; Dietrich et al., 2017; Zelesco et al., 2018). Finally, mean and SD of the selected 5 data were calculated for Young’s modulus and SWV of the ROI.

### Statistical Analysis

The interobserver agreement was analyzed by calculating intraclass correlation coefficient (ICC) and defined as poor if ICC was <0.4, moderate if it was 0.41–0.6, good if it was 0.61–0.79, and excellent if it was >0.8. The agreement of SWS between the observers was evaluated additionally with Bland – Altman analysis (Giavarina, 2015). The mean of difference (bias) and the 95% confidence interval for bias were calculated. The bias between the observers was evaluated for significant difference (*P* < 0.05) from zero error with a one sample *t*-test. The difference in SWS for the three technical factors (measuring site, approach, and depth) on abdominal organs (liver, spleen, kidney, prostate, and pancreas) was evaluated. The normality of SWS in each SWE examinations was assessed using Shapiro–Wilk test. Then, for data of normal distribution, the independent *t*-test was used to compare the SWS according to the technical factors. For data of non-normal distribution, the Mann–Whitney *U* test was used. Wilcoxon signed rank test was used to determine the difference in SWS in the spleen according to anesthesia. One-way analysis of variance and multiple comparison with the Scheffe’s correction was used to compare SWS between lymph nodes. To investigate the reliability of measurements according to different technical factors in the liver, spleen, kidney, prostate, and pancreas, the difference in SD of the SWS was compared using Mann-Whitney *U* test. *P*-value less than 0.05 was considered as statistically significant and *P* less than 0.001 as highly significant. Data of normal distribution were presented as mean with SD and data of non-normal distribution were presented as the median ± interquartile range. Statistical analysis was performed by one veterinarian (JJ) using commercial software (IBIM SPSS Statistics 21, IBM Corp., New York, United States).

## Results

A total of 27 SWE examinations in individual male dog and 25 examinations in female dogs (excluding the prostate) were performed by each examiner. SWS was measured successfully in 12 organs. In the renal medulla, proper propagation could not be obtained from both kidneys, thus renal medulla was excluded from further analysis.

The ICCs and Bland – Altman analysis of abdominal organs according to the technical factors are listed in Table 2. Reproducibility was excellent in the liver (0.864–0.948), except for the left liver lobe at 10–15 mm depth with intercostal approach (0.165). All SWEs in other abdominal organs showed good to excellent interobserver agreement (0.603–0.871), but the right prostate showed moderate reproducibility. For the SWS of all abdominal organs, the bias between observer was not significantly different from zero. The ICCs of lymph nodes showed good to excellent reproducibility (0.738–0.994) except for the axillary lymph node that showed moderate reproducibility and significant difference in bias from zero (*P* = 0.031; Table 3). The salivary gland and thyroid had excellent and good interobserver agreement, respectively.

**TABLE 2:** Interobserver agreement and Bland – Altman analysis in two-dimensional shear wave elastography in abdominal organs according to technical factors.

					Bland-Altman analysis (kPa)	
					
Organs	Sites	Approach	Depth (mm)	Intraclass correlation coefficient	Mean bias (95% CI bias)	*P*-value
Liver	Right	Subcostal	5–10	0.922	0 (0.98, −0.98)	1
			10–15	0.890	−0.3 (0.89, −1.49)	0.176
		Intercostal	5–10	0.912	−0.14 (1.61, −1.89)	0.646
			10–15	0.884	−0.05 (1.07, −1.17)	0.782
	Left	Subcostal	5–10	0.864	0.17 (1.66, −1.33)	0.528
			10–15	0.948	0.09 (1.01, −0.83)	0.590
		Intercostal	5–10	0.871	0.26 (1.31, −0.80)	0.19
			10–15	0.165	0.3 (1.87, −1.28)	0.297
Spleen	–	–	0–5	0.861	−0.17 (2.11, −2.45)	0.733
			5–10	0.670	−0.11 (1.77, −2.00)	0.733
Renal cortex	Right	–	–	0.839	−0.09 (0.43, −0.60)	0.347
	Left	–	–	0.819	−0.01 (0.84, −0.86)	0.964
Prostate	Right	–	–	0.534	0.20 (2.62, −2.23)	0.688
	Left	–	–	0.871	0.04 (1.91, −1.82)	0.909
Pancreas	Right	–	–	0.858	−0.03 (1.00, −1.06)	0.873
	Body	–	–	0.603	−0.35 (0.85, −1.55)	0.123

*The interobserver agreement in the spleen in this table is estimated before anesthesia.*

**TABLE 3:** Interobserver agreement and Bland – Altman analysis in two-dimensional shear wave elastography in the lymph nodes, salivary gland and thyroid.

		Bland-Altman analysis (kPa)	
		
	Intraclass		
	correlation	Mean bias	
Organs	coefficient	[95% CI bias] (kPa)	*P*-value
Submandibular lymph node	0.968	0.03 (0.88, −0.82)	0.852
Retropharyngeal lymph node	0.738	0.34 (1.58, −0.89)	0.141
Axillary lymph node	0.552	0.41 (1.33, −0.51)	0.031*
Medial iliac lymph node	0.815	0.12 (1.61, −1.38)	0.659
Inguinal lymph node	0.994	−0.3 (0.72, −1.32)	0.123
Submandibular salivary gland	0.889	0.01 (1.59, −1.57)	0.968
Thyroid	0.723	0.26 (2.16, −1.64)	0.442

*P < 0.05.

**FIGURE 4:**
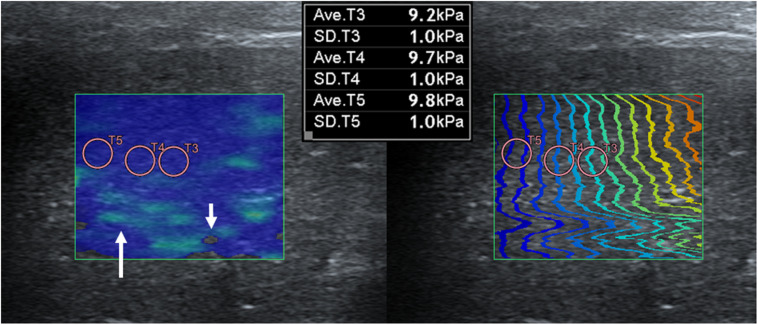
Elastographic images in the liver. Near field, the region at 5–10 mm depth from the liver capsule, has uniform blue color in the color map and contour lines with constant intervals in the propagation map. Far field, the region at 10–15 mm depth from the liver capsule, appeared heterogeneously in the color map with focal green color (long arrow) and block spots (short arrow) presenting signal void and distorted contour lines in the propagation map.

Shear wave elastography examination of the liver was possible without compression by the probe, regardless of the depth, approach, and measuring sites. In the elastographic images of the liver, uniform blue color in the color map and parallel contour lines in the propagation map were obvious in the near FOV field (5–10 mm depth). However, in the far FOV field (10–15 mm depth), multifocal distribution of green spots in the color map, distorted contour lines in the propagation map, or presence of black was found (Figure 4). The SWS of the liver was not significantly different according to the measuring site (*P* = 0.489–0.965) and approach (*P* = 0.596–0.965). The SWS of the liver tend to be higher at the far field than at the near field (Table 4). Significant difference of SWS according to ROI depth was found in the left liver with intercostal scan by observer 1, but no significant difference by observer 2. SD of SWS was not significantly different according to the measuring site, approach, and depth.

**TABLE 4:** Young’s modulus (kPa) and shear wave velocity (m/s) in the liver according to the measuring site, approach, and depth from liver capsule.

		Depth		
		
Measuring				
site	Approach	5–10 mm	10–15 mm	*P*-value
Right	Subcostal	7.7 ± 0.36 kPa	8.45 ± 1.36 kPa	0.222
		1.63 ± 0.03 m/s	1.69 ± 0.11 m/s	
	Intercostal	7.57 ± 1.17 kPa	8.36 ± 1.16 kPa	0.133
		1.60 ± 0.11 m/s	1.67 ± 0.11 m/s	
Left	Subcostal	7.61 ± 1.29 kPa	8.27 ± 1.32 kPa	0.305
		1.60 ± 0.13 m/s	1.67 ± 0.14 m/s	
	Intercostal	7.8 ± 0.06 kPa	8.1 ± 0.38 kPa	0.019
		1.62 ± 0.1 m/s	1.64 ± 0.03 m/s	

*Data of normal distribution are presented as mean with SD and data of non-normal distribution are presented as the median ± interquartile range.*

Shear wave elastography in the spleen before anesthesia was easily and reliably performed showing uniform blue color and constant interval of contour lines in the elastographic images. After anesthesia, the splenic parenchyma showed green to blue changes in the color map and SWS was significantly increased (*P* = 0.011; Table 5). SWS recovered to the pre-anesthetic SWS levels during the recovery phase (*P* = 0.028). SWS in the spleen tended to be higher at the far field (5–10 mm depth) than that at the near field (0–5 mm depth), however, there was no significant difference (*P* = 0.387). No statistical difference in SD of SWS was found according to anesthesia and ROI depth.

**TABLE 5:** Mean value of Young’s modulus (kPa) and shear wave velocity (m/s) in the spleen according to anesthesia.

	Young’s modulus (shear wave velocity)		
	
Depth (mm)	Pre-anesthesia	Anesthesia phase	Recovery phase
0–5	8.97 ± 1.3	14.79 ± 3.21*	9.86 ± 3.05
	(1.73 ± 0.13)	(2.22 ± 0.26)	(1.80 ± 0.26)
5–10	9.35 ± 1.82	14.91 ± 2.88*	10.18 ± 2.88
	(1.77 ± 0.17)	(2.23 ± 0.24)	(1.85 ± 0.24)

Data are presented as mean and standard deviation. *P < 0.05.

**FIGURE 5:**
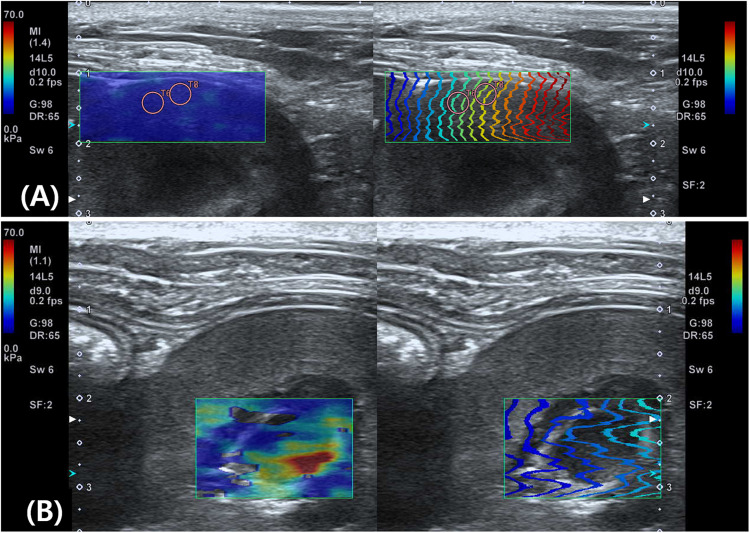
Elastographic images of the kidney. On shear wave elastography (SWE) of the renal cortex **(A)** shows homogeneous blue color in the color map and constant contour lines in the propagation map, whereas SWE of the renal medulla **(B)** shows heterogeneity with various colors and severe distortion of contour lines in the propagation map.

In conventional ultrasonography of the kidney, the sagittal image of the left kidney was visualized clearly without compression, whereas it was more difficult to visualize the right kidney without compression because it is deep-seated and located in the upper abdomen. Thus, the intercostal approach was inevitable for the right kidney in three dogs. In both the left and right kidneys, SWE measurement was easily conducted with uniform blue color and constant contour line in the renal cortex. Whereas the color map in the renal medulla was severely heterogeneous and contour lines in the propagation map were distorted, a reliable measurement of SWS was not possible (Figure 5). The SWS was significantly higher in the right renal cortex than in the left renal cortex [right; 8.37 ± 1.11 kPa (1.67 ± 0.1 m/s), left; 6.67 ± 0.91 kPa (1.5 ± 0.09 m/s)] (*P* < 0.001). SD of SWS was significantly higher in the right renal cortex than in the left (*P* = 0.03).

Shear wave elastography was easily conducted in the pancreas in both measuring sites with uniform blue color mapping and constant contour line. SWS was significantly higher in the pancreatic body than in the right pancreatic lobe [body; 7.78 ± 0.89 kPa (1.62 ± 0.09 m/s), right; 6.4 ± 0.93 kPa (1.47 ± 0.09 m/s)] (*P* < 0.001). There was no significant difference in SD of SWS (*P* = 0.489) according to the measuring sites.

Although it was challenging to visualize the prostate without compression because of its location in the pelvic cavity, shear wave was properly induced for the visible part of the prostate. No significant difference was found in SWS [right; 8.72 ± 1.53 kPa (1.7 ± 0.15m/s), left; 8.41 ± 1.59 kPa (1.67 ± 0.15 m/s)] (*P* = 0.828) and SD of SWS (*P* = 0.383) between the left and right prostate.

**FIGURE 6:**
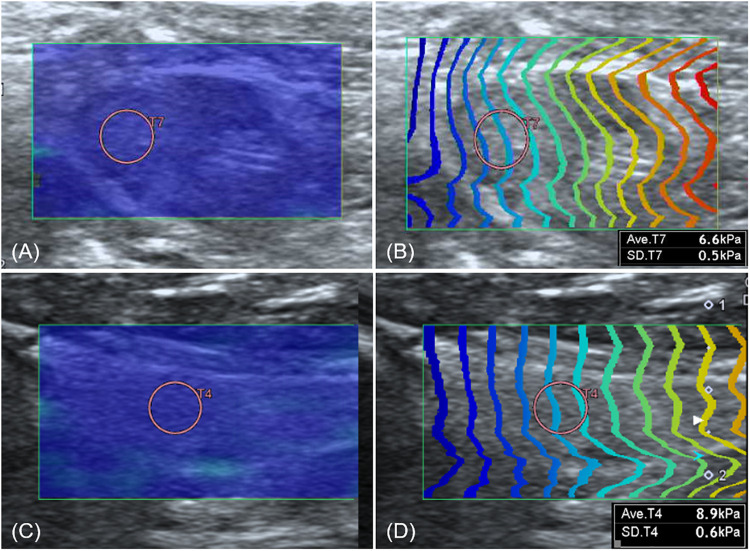
Elastographic images of the axillary lymph node **(A,B)** and thyroid **(C,D)**. Both the axillary lymph node and thyroid show uniform blue color in the color map **(A,C)** and constant intervals of contour lines in the propagation map **(B,D)**.

Shear wave of all lymph nodes, submandibular salivary gland, and thyroid was easily induced without compression, shown as uniform blue color in a color map and equal interval of contour lines (Figure 6). The SWS in the lymph nodes, submandibular salivary gland and thyroid are presented in Table 6. SWS was not significantly different between lymph nodes (*P* = 0.142).

**TABLE 6:** Young’s modulus (kPa) and shear wave velocity (m/s) in the lymph nodes, salivary gland, and thyroid.

	Young’s modulus	Shear wave
Organs	(kPa)	velocity (m/s)
Submandibular lymph node	7.84 ± 0.71	1.62 ± 0.07
Retropharyngeal lymph node	7.29 ± 1.07	1.57 ± 0.1
Axillary lymph node	6.92 ± 0.48	1.52 ± 0.07
Medial iliac lymph node	7.92 ± 0.96	1.63 ± 0.09
Inguinal lymph node	7.18 ± 1.06	1.55 ± 0.1
Submandibular salivary gland	10.07 ± 1.38	1.83 ± 0.12
Thyroid	7.22 ± 1.02	1.57 ± 0.1

*Data of normal distribution are presented as mean with SD and data of non-normal distribution are presented as the median ± interquartile range.*

## Discussion

### Feasibility and Reproducibility of 2D SWE in Canine Soft Tissue Organs

Two-dimensional shear wave elastography was feasible for acquisition of elastographic images and reliable SWS values from canine soft tissue organs. 2D SWE was performed with excellent interobserver agreement (over 70%) among 25 measurements in 12 organs. High reproducibility was possible in virtue of motion control during SWE and screening of the data collection. Gentle breathing and breath holding before shear wave induction are recommended in humans, because a higher stiffness value can be obtained at end-expiration than end-inspiration (Ling et al., 2013; Barr et al., 2015a; Sigrist et al., 2017; Zelesco et al., 2018). In a canine study performed by Holdsworth et al. (2014) using pSWE in the liver, spleen, and kidneys, it was hard to obtain reliable data and examinations were repeated many times owing to poor cooperation and panting of the dogs. So, they suggested sedation or general anesthesia to reduce the motion effect on SWE in dogs (Holdsworth et al., 2014). In our study, anesthesia allowed us to reduce movement and respiration, to obtain B-mode images without compression by the probe, and to maintain the probe perpendicular to the target organ for optimizing the displacement of tissue, limiting push pulse refraction (Shiina et al., 2015; Barr et al., 2015a; Sigrist et al., 2017; Zelesco et al., 2018).

A guideline for human elastography recommends both selection of data of which the interquartile range/median ratio of SWS less than 0.3 or SD less than 20% of the SWS inside ROI and a minimum of three SWS measurements as reliable criterion (Barr et al., 2015a; Dietrich et al., 2017; Zelesco et al., 2018). We followed the modified guideline more strictly to improve SWE reliability; data with SD less than 15% of the mean value were selected and the mean value of 5 data was used.

This study estimated SWV and Young’s modulus on 2D SWE. In SWE, shear waves are generated by acoustic radiation force and propagate perpendicularly to the ultrasound with a speed of approximately less than 30 m/s. SWE does not measure the stiffness itself but it can evaluate tissue stiffness by measuring the shear waves propagation speed. SWS can be used to estimate the tissue shear modulus. For a purely elastic and isotropic medium, shear modulus (G) is directly related to the SWV (c_s_) and can be calculated by a simple mathematical equation as G = ρc_s_^2^ where ρ is the biological tissue density. Young’s modulus (E) is calculated using equation as E = 3G with assumption of constant density, homogeneity, isotropy, linear elasticity, and incompressibility (Shiina et al., 2015). However, this assumption does not correspond to soft tissue (Mo et al., 2016; Lin et al., 2017; Rominger et al., 2018; Davis et al., 2019). For example, the assumption about local homogeneousness in the examined tissue may be more invalid in organs with thin layer structures such as myocardium and bladder wall compared to in large his study estimated SWV and Young’s modulus on 2D SWE l organs such as the liver, because SWS is affected by the thickness of the thin layers as well as the mechanical property of the tissue (Mo et al., 2016). Similarly, Young’s modulus is inaccurate in the musculoskeletal system which comprises viscoelastic, heterogeneous, and anisotropic tissue (Davis et al., 2019). In a study about spatial dependent mechanical properties of the heel pad using SWE, Young’s modulus was not used considering the heterogeneity of the tissue (Lin et al., 2017). Thus, SWE should be used to assess the changes in stiffness of the tissue but not to quantify stiffness itself (Chatzistergos et al., 2018).

### Effects of the Technical Factors on SWS Measurement in Abdomen

The effects of the technical factors on SWS were assessed based on the difference in SWS and SD according to the measuring site, approach and ROI depth in the liver, ROI depth and anesthesia in the spleen and measuring site in the renal cortex, pancreas and prostate. Probe positioning and user operation also can affect SWE (Rominger et al., 2018). However, in a study about SWE of the supraspinatus tendon and muscle, errors due to repositioning and user operation were small and high intra-user and inter-user repeatability, and high day-to-day repeatability were achieved (Baumer et al., 2017). So, in our study, probe repositioning and repeatability were not assessed.

In the liver, all measurements regardless of the measuring sites, scan approach, and FOV depth had excellent reproducibility. However, SWE performed at the far field in the left liver lobe with intercostal scan showed poor reproducibility. In humans, the right liver lobe with intercostal approach is recommended for liver elastography, because it is far from the heart and the entire lobe is easier visualized with the intercostal approach than the subcostal approach (Cosgrove et al., 2013; Ling et al., 2013; Ferraioli et al., 2015; Guimarães et al., 2020). Considering that beagle dogs have deep chest and the liver is located in upper abdomen, close positioning of the left liver lobe to the heart may contribute to poor reproducibility. In this study, SWE was performed in dogs after fasting to avoid the effect of the gastric dilation like the recommendation for liver elastography in human (Ferraioli et al., 2015). In 2D SWE, FOV provides a cumulative data that is the average of stiffness at several points, FOV should be large enough to provide consistent measurements in the liver (Ferraioli et al., 2015; Guimarães et al., 2020). In our study, a 3 × 3 cm FOV size was used to represent the heterogeneous stiffness in the liver (Ferraioli et al., 2015; Guimarães et al., 2020). The SWS of liver in this study was comparable with the result of a previous study that performed 2D SWE in the liver in healthy dogs (Tamura et al., 2019a). In the study, SWS of the right lobe (6.93 ± 0.79 kPa) was significantly higher than that of the left lobe (6.02 ± 0.61 kPa). They considered that the differences in liver stiffness between sides could be related with the influence of the movement of the heart, lungs, diaphragm, and stomach on left lobe during SWE. However, in our study, SWS of the liver was not significant different according to measurement site (between left and right lobes) and approach (between intercostal and subcostal) the liver. Although the previous study performed 2D SWE using the ultrasound machine manufactured by the same company (Canon medical system) as ours, direct comparison of the results was difficult because different ultrasound model (Aplio 500 vs 600) and probe (convex vs linear) were used. In human literature, the linear probe derived a higher SWS of the liver than convex probe in hepatic cirrhosis (Ferraioli et al., 2018).

We assessed depth effect on SWE in liver and spleen. Human literature recommends that SWE is performed minimally 10 mm below the liver capsule, because physiologic fibrosis in the liver capsule can induce false increase in the SWS in the subcapsular region and inaccurate measurement because of reverberation artifacts (Barr et al., 2015a; Dietrich et al., 2017; Nadebaum et al., 2018; Zelesco et al., 2018). In the spleen, SWS is measured at least 10 mm below the spleen capsule (Ferraioli et al., 2014). However, the physiologic effect of the liver capsule and splenic capsule on SWE measurement and the recommendation of ROI depth from them in 2D SWE have not reported in dogs. In our study, the depth effect was assessed based on the commonly used depth for the liver and spleen in clinic. In liver, SWS tended to be higher in the far field than in the near field with showing a heterogeneous color map and a distorted propagation map. This result is comparable with a human study using the same equipment as ours (Gress et al., 2019). However, in a previous canine study using pSWE, ROI depth had a significantly negative relationship with SWS (Holdsworth et al., 2014). The effect of depth is variable according to the ultrasound unit, elastographic technique, probe and even the software version used, which makes direct comparisons difficult (D’Onofrio et al., 2010; Tamura et al., 2019b). In a human study, heel pad stiffness was measured at 8 depths using 2D SWE and spatial dependent mechanical property of tissue was proposed that the stiffness of the heel pad was the highest nearest to the plantar skin and decreased continuously with increasing depth (Lin et al., 2017). However, it is difficult to describe spatial dependent property of liver in our study, because SWS was measured at only two depths (5–10 mm vs 10–15 mm). Meanwhile, we measured the SWE below 10 mm of the liver capsule to avoid false increase of SWS by physiologic fibrosis, and since FOV contained only homogenous hepatic parenchyma which composed of hepatocyte, the depth related effect of spatial dependent mechanical property on the SWS of the liver may not be high in this study. Rather, because the signal to noise ratio can be reduced according to depth in 2D SWE, (Cosgrove et al., 2013) increased heterogeneity in elastographic images in the far field and generation of block spots is possible, resulting in higher SWS in the far field than in the near field. However, effect of ROI depth was questionable because significant difference of SWS was found only in the left liver with intercostal scan which have both poor interobserver agreement in 10–15 mm depth and different significant difference between observer 1 and 2. Thus, the difference of SWS according to ROI depth is not considered to be significant, similar to spleen.

In the spleen, proper shear wave could be induced without difficulty even in conscious dogs, owing to the superficial location of the spleen, similar with the study performed by Tamura et al. (2019a). Anesthesia is not usually needed for most ultrasound examinations, but sedation or general anesthesia can be used to reduce the motion effect for accurate and consistent data on SWE in dogs (Holdsworth et al., 2014). In our study, the effect of anesthesia on the SWE was investigated in the spleen because the spleen becomes easily enlarged due to congestion after anesthesia in dogs. The SWS of spleen was significantly higher than right hepatic lobe, and they suggested fibroelastic supporting tissue and fine reticulum in the spleen as the cause of higher stiffness of the spleen. This result was similar to our study, although the SWS of the spleen was lower than that of Tamura’s study. After anesthesia, SWS in the spleen increased significantly and it can be related with congestion caused by our anesthetic protocol using medetomidine. Medetomidine, an alpha-2 agonist, affects blood pressure and flow and can induce vasodilation (widening of blood vessels) and splenic congestion (swelling; Wilson et al., 2004). This result is similar to human studies showing that stiffness in the hepatic congestion was increased (Barr et al., 2015a; Dietrich et al., 2017; Sigrist et al., 2017; Nadebaum et al., 2018; Zelesco et al., 2018).

**FIGURE 7:**
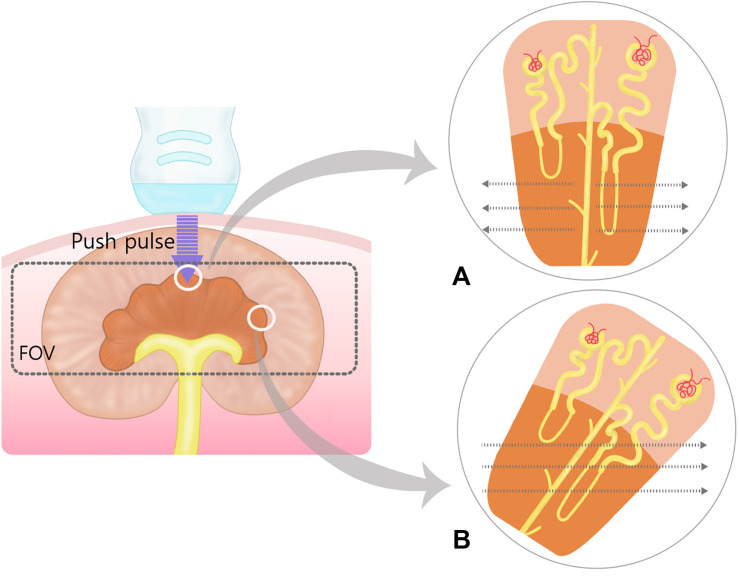
Schematic representation of the push pulse and shear wave in the renal medulla. Within field of view (FOV), the shear waves (dotted arrows) propagate perpendicular to the push pulse. **(A)** When push pulses are sent parallel to the renal medulla, shear waves propagate orthogonally to the loops of Henle and collecting ducts at lower speeds due to more biological interfaces. **(B)** In oblique manner, shear waves will travel at higher speed than in **(A)** because of less biological interfaces. The oblique and perpendicular anisotropy within the renal medulla results in heterogenous shear wave speed within the field of view.

In SWE of the kidney, anatomic anisotropy of the kidney parenchyma can affect shear waves propagation speed and result the inconsistent SWE values (Gennisson et al., 2012; Leong et al., 2020). In our study, SWE in the renal medulla was unreliable because of its heterogeneity in the color and propagation maps. The renal pyramid, in the renal medulla, consists of the loops of Henle, and collecting ducts, which have parallel orientation from the renal crest to the renal capsule (Evans and De Lahunta, 2013). When push pulses are sent parallel to these linear structures, shear waves propagate orthogonally to them, creating interfaces that decrease SWS. Conversely, when push pulses are sent orthogonally to these linear structures, shear waves propagate parallel to them, without interfaces, increasing SWS (Sigrist et al., 2017). We visualized the sagittal view of the kidney but not all renal pyramids could be parallel to the probe and the direction of shear wave could be oblique to some renal pyramids (Figure 7). The oblique orientation of shear waves to the renal pyramid may have contributed to the heterogeneity of elastographic images. In a pSWE study, there was no difference between the right and left renal cortex in dogs and cats (Holdsworth et al., 2014; Garcia et al., 2015) and also in human (Asano et al., 2014). In a study about 2D SWE of normal kidney and various degree of chronic kidney diseases, there was no statistical difference between both kidney (Grosu et al., 2019). However, another study about SWE in the renal allograft presented urinary pressure, hydronephrosis vascular perfusion, body score index, and probe pressure as factors affecting SWE measurements (Early et al., 2017). In our study, right renal cortex had significantly higher SWS than left side and it may be related to the slight compression applied by the probe to the right kidney due to its anterior and deeper location. In human, both pSWE and endoscopic ultrasound elastography are sensitive to distinguish pancreatic cancer, acute pancreatitis, and chronic pancreatitis from healthy parenchyma, albeit with low specificity (Cosgrove et al., 2013; Goertz et al., 2016). In this study, 2D SWE allowed to evaluate the right pancreatic lobe and pancreatic body with reliable interobserver agreement and qualitative elastographic images. A significant difference in SWS between the pancreatic body and the right pancreatic lobe was observed. The effect of the measuring site on SWS in the pancreatic region is controversial in human studies using pSWE (Stumpf et al., 2016; Zaro et al., 2016); one study found a difference in SWSs in the pancreatic head, body, and tail and considered that the differences in SWS were because of the differences in the ROI angle and depth between each pancreatic region (Ferraioli et al., 2015). It is difficult to compare between the J shaped human pancreas, which consists of the head, body and tail extending from the duodenum to the spleen in the upper abdomen, and the triangular-shaped canine pancreas, which extends to both left and caudal directions (Evans and De Lahunta, 2013; Ferraioli et al., 2015). In our study, the probe could be perpendicular to both the pancreatic body and the right pancreatic lobe and both pancreatic regions were located just below the skin surface, thus the differences in SWE cannot be attributed to the FOV angle and ROI depth. We considered probe compression could be one technical factor to account for the higher SWS in the pancreatic body compared with the right pancreatic lobe, because of the anatomical location of pancreatic body below the pylorus and the effort to scan the portal vein dorsally to the pancreatic body as a landmark in ultrasound (Evans and De Lahunta, 2013; Penninck and d’Anjou, 2015). The left pancreatic lobe was excluded from this study because it was difficult to scan without compression owing to its caudal position to the stomach.

In humans, 2D SWE was superior to conventional ultrasound, digital rectal examination and prostate-specific antigen assay to differentiate between benign prostatic lesion and malignancy and showed high sensitivity and specificity (Barr et al., 2017; Sang et al., 2017). For the optimal prostate elastography in human, minimal preload over the prostate is an important factor because of anatomic location of the prostate and the intra-rectal approach. So, the left lateral position with bended knees and hip flexion or the lithotomy position is recommended for the prostate elastography. The prostate is located in the pelvic cavity in in dogs and the whole prostatic parenchyma could not be visualized with the abdominal approach because of the pelvic bone. However, qualitative elastographic images were acquired in the visible part of the prostate through abdominal approach with the dog in dorsal recumbency and SWS could be quantified from both prostatic lobes, similar to pSWE (Feliciano et al., 2015). There was no significant difference in stiffness between lobes, consistent with a previous pSWE study in dogs (Garcia et al., 2015). The adjustment of FOV size is recommended to include the prostate capsule and periprostatic tissues and take care of excluding the bladder. Similarly, we adjusted FOV size to fill the prostate at least 80% in the FOV. In addition, the center location of the area of the stiff lesion within the FOV and the ROI encompassing only the lesion are better for SWE in the prostate (Correas et al., 2015; Barr et al., 2017).

Dogs have been used as animal models for human elastography studies in the liver, kidney, pancreas, prostate, and thrombus (Bhutani et al., 2009; Rivero-Juárez et al., 2012; Correas et al., 2015; Gao et al., 2015; Sun et al., 2017). In this study, the technical factors which affected 2D SWE measurements in dogs were similar with those in humans such as ROI depths and site. However, dogs and human have differences in organ geometry, depth and acoustic clutter environments which can generate unequal variabilities in shear wave generation, shear wave propagation and ultrasonic tracking. Thus, we have regard to the difference in two species without replacement our study for variability study in human subjects.

### 2D SWE in the Lymph Nodes, Salivary Gland and Thyroid

The feasibility of strain elastography was evaluated for the assessment of thyroid stiffness, but neither 2D SWE nor pSWE have been studied for the submandibular, retropharyngeal, and axillary lymph nodes, salivary gland, and thyroid in veterinary medicine (Lee et al., 2015). All lymph nodes, salivary gland and thyroid had reliable interobserver agreement and were visualized with uniform color map and constant contour lines on 2D SWE without compression in our study.

In human, the inter-observer and intra-observer repeatability of 2D SWE of the thyroid are high (Cosgrove et al., 2017; Kyriakidou et al., 2018). Similar to the prerequisite in other organs, pre-compression, the ROI size and positions with avoiding the heterogeneous areas related with gross calcifications, cyst, or artifacts should be considered during SWE of the thyroid (Cosgrove et al., 2017; Kyriakidou et al., 2018). 2D SWE has good sensitivity and specificity for identification of thyroid nodules and showed comparable result with pSWE in differentiating between malignant and benign nodules (Kyriakidou et al., 2018; Zhao and Xu, 2019).

In a previous canine study regarding identification of lymph node metastasis, with ultrasonography, in dogs with mammary tumor, axillary and inguinal lymph nodes with metastasis were significantly stiffer than normal and benign lymph nodes (Silva et al., 2018). The sensitivity and specificity for metastasis identification was superior for 2D SWE than for B-mode and Doppler evaluation, recording 100% sensitivity, 94% specificity in axillary lymph node and 95% sensitivity, 87% specificity in the inguinal lymph nodes (Silva et al., 2018). In humans, a combination of conventional ultrasound and elastography is recommended to increase the sensitivity and specificity for the diagnosis of lymph node malignancy (Cosgrove et al., 2013; Sigrist et al., 2017). In addition, maximum Young’s modulus of ROI was suggested in 2D SWE for lymph node compared to the mean Young’s modulus for identifying the focal cortical metastases or necrotic areas in the node and SWS was measured from the stiffest region within the lymph node based on the color-coded elastogram (Choi et al., 2013; Lo et al., 2019).

There are only a few reports about SWE of the salivary gland in human (Mantsopoulos et al., 2015; Arslan et al., 2020). In one study about the salivary gland elastography, there was no significant effects of gender, smoking and measuring sites on the SWS of the parotid and the submandibular gland (Mantsopoulos et al., 2015). However, the SWE values of both salivary glands were increased significantly according to pre-compression with the ultrasound probe (Mantsopoulos et al., 2015). Further studies are required to evaluate the technical factors affecting the 2D SWE of the thyroid, lymph nodes, and salivary glands in dogs and verify the usefulness of 2D SWE in clinically affected dogs with conditions such as multicentric lymphomas, hypothyroidism, and salivary gland tumors.

### Limitation and Further Study

There are some limitations in this study. First, healthy organs were not confirmed with cytological or histological examination but presumed by physical examination, blood test, urinalysis, radiography, and ultrasonography in dogs. Second, this study was performed in a small number of beagles of the same breed, similar body size and uneven male and female number. Because a previous canine pSWE study showed that sex and body weight had a significant effect on SWS in specific organs, (Cosgrove et al., 2013) further studies examining the effect of breed, weight and sex on SWS in 2D SWE are required. Third, there is no clear recommendation in dogs regarding the preferred the depths of measurement in the liver and spleen. We selected the depths of measurement commonly used for ultrasonography of the liver and spleen in dogs. Fourth, the intra-observer reliability was not assessed in this study. High intra-observer reliability using 2D SWE was reported in the liver and spleen in healthy dogs (Tamura et al., 2019a). However, intra-observer reliability of 2D SWE should be assessed in various soft tissue organs further.

## Conclusion

In conclusion, evaluation of stiffness in abdominal organs (liver, spleen, renal cortex, pancreas, and prostate), lymph nodes (medial iliac, inguinal, axillary, submandibular, and retropharyngeal lymph nodes), submandibular gland and thyroid was feasible with 2D SWE, in anesthetized dogs with high reproducibility. However, 2D SWE was not suitable for assessing stiffness in the renal medulla. The effect of scan sites and approach, ROI depth, and anesthesia were investigated in abdominal organs. In liver and spleen, SWS tended to be higher in the far field than in the near field, but no significant difference. Measuring site in the pancreas and renal cortex, anesthesia in the spleen had significant effect on SWS. Significant differences in SWS according to depth in the liver, measuring site in the pancreas and renal cortex, and anesthesia in the spleen were observed. Therefore, these factors should be considered during SWS measurement in 2D SWE. This study provides basic data for further studies on 2D SWE on pathological conditions that may increase tissue stiffness in dogs.

## Data Availability Statement

All datasets presented in this study are included in the article/supplementary material.

## Ethics Statement

The animal study was reviewed and approved by the Institutional Animal Care and Use Committee of Chonnam National University (CNU IACUC-YB-2018-90).

## Author Contributions

Hypothesis and experimental design was generated by J-WJ and JC. SWE was performed by J-WJ and HJ with support by S-KL and YJ. J-WJ performed statistical analysis. The manuscript was written by J-WJ and revised by JC. All authors contributed to the article and approved the submitted version.

## Conflict of Interest

The authors declare that the research was conducted in the absence of any commercial or financial relationships that could be construed as a potential conflict of interest.
